# NEK7 Promotes Pancreatic Cancer Progression And Its Expression Is Correlated With Poor Prognosis

**DOI:** 10.3389/fonc.2021.705797

**Published:** 2021-07-06

**Authors:** Zilong Yan, Jianhua Qu, Zhangfu Li, Jing Yi, Yanze Su, Qirui Lin, Guangyin Yu, Zewei Lin, Weihua Yin, Fengmin Lu, Jikui Liu

**Affiliations:** ^1^ Department of Hepatobiliary Surgery, Peking University Shenzhen Hospital, Shenzhen-Peking University-Hong Kong University of Science and Technology Medical Center, Shenzhen, China; ^2^ Department of Hepatobiliary Surgery, Peking University Shenzhen Hospital, Shenzhen, China; ^3^ Department of Pathology, Peking University Shenzhen Hospital, Shenzhen, China; ^4^ Department of Microbiology and Infectious Disease Center, School of Basic Medical Sciences, Peking University Health Science Center, Beijing, China

**Keywords:** NEK7, pancreatic ductal adenocarcinoma, adhesion, prognosis, migration, invasion

## Abstract

The prognosis for pancreatic ductal adenocarcinoma (PDAC) patients is still dismal. Elucidation of associated genomic alteration may provide effective therapeutic strategies for PDAC treatment. NIMA-related protein kinase 7 is widely expressed in various tumors, including breast cancer, colorectal cancer and lung cancer, and promotes the proliferation of liver cancer cells *in vitro* and *in vivo*. We investigated the protein expression level of NEK7 in tumor tissues and adjacent normal tissues using immunohistochemistry of 90 patients with PADC. Meanwhile, the RNA expression level of NEK7 was examined using database-based bioinformatic analysis. Correlation and significance of NEK7 expression with patient clinicopathological features and prognosis were examined. Cell proliferation, cell adhesion, migration and invasion capabilities were measured following downregulation of NEK7 expression. 3D tumor organoids of pancreatic cancer were established and splenic xenografted into nude mice, then liver metastatic ability of NEK7 was evaluated in following 4 weeks. We observed NEK7 expression was upregulated in tumor tissues compared to normal tissues at both RNA and protein levels using bioinformatic analysis and immunohistochemistry analysis in PDAC. NEK7 expression was undetectable in normal pancreatic ducts; NEK7 was overexpressed in primary tumor of PDAC; NEK7 expression was highly correlated with advanced T stage, poorly differentiated histological grade invasive ductal carcinoma, and lymphatic invasion. Meanwhile, patients with higher NEK7 expression accompanied by worse survival outcome. Moreover, NEK7 promoted migration, invasion, adhesion, proliferation and liver metastatic ability of pancreatic cancer cells. Taken together, our data indicate that NEK7 promotes pancreatic cancer progression and it may be a potential marker for PDAC prognosis.

## Introduction

Pancreatic ductal adenocarcinoma (PDAC) is the fourth leading cause of cancer death with a poor 5-year survival rate less than 10% (1). Due to early metastasis and little opportunity for curable surgery (2), the mortality to incidence ratio of PDAC is 94% (3). Pancreatic cancer patients are usually diagnosed at terminal stages with distant metastases, the outcome remains poor with a 5-year survival rate of just 2.9% (4). Since limited benefits are demonstrated with traditional treatments like surgical resection and chemotherapy, it is essential to further understand the mechanisms underlying genomic alteration and explore non-surgical therapeutic approaches for effective treatment of PDAC.

The NIMA Related protein kinase family (NEKs) includes 11 members which were originally identified as human orthologs to the Aspergillus nidulans protein kinase, NIMA (Never In Mitosis, gene A) kinase (5, 6). In particular, The NIMA related protein kinase family is involved in regulating cell cycle and mitotic progression (7–9). Of these NEK family members, NEK7 is the smallest protein, composed of only a catalytic domain with a 30–40 amino acid N-terminal extension, which shares more than 85% sequence identity to NEK6 (10, 11). Previous studies showed that overexpression of NEK6 was detected in a range of human tumor types, including breast, lung, liver, gastric and colorectal cancers (12–16). Biological roles of NEK6, such as interaction with HIF-1a, premature senescence suppression, cell proliferation promoting were then demonstrated (17–20). On the other hand, NEK7 is widely expressed in various tissues, such as the heart, liver, lung, brain, muscle, testis, leukocyte, and spleen (11). Accumulating evidence suggests that NEK7 is involved in mitosis regulation through an intricate mechanism (21–24). Moreover, NEK7 is significantly increased in squamous cell carcinoma of the head and neck (25), breast cancer (26), colorectal cancer and lung cancer (27), and promotes the proliferation of liver cancer cells *in vitro* and *in vivo*, due to its significant relationship with Ki67 expression in HCC tissues (28). However, the function of NEK7 in PDAC remains unknown.

In present study, we showed for the first time, the differential expression pattern of NEK7 in PDAC tissues and cells. We investigated expression level and biological significance of NEK7 using database based bioinformatic analysis, clinical samples from patients with PDAC and cultured PDAC cells. Bioinformatic and clinicopathological analysis revealed a positive correlation between NEK7 expression to advanced T stage, lymphatic invasion and histological grade in PDAC. Our results proved that NEK7 showed a prognostic significance in survival of patients with PDAC. We detected a significant involvement of NEK7 in the migratory, invasive and adherent capacities of pancreatic cancer cells. *In vivo* experiments revealed that downregulation of NEK7 expression inhibited cancer cell liver metastases. Taken together, these results suggest that NEK7 might contribute tumor progression and prognosis of PDAC. Therefore, NEK7 is a potential target for treatment of PDAC.

## Materials and Methods

### Pancreatic Cancer Tissues and Ethical Approval

PDAC samples and tumor-adjacent normal tissues collected from our institution were used to study NEK7 expression. Tissues were embedded, sliced and stained, and sections were observed using an optical microscope (BX53, Olympus, Japan). For The clinicopathological characteristics analysis, the human tissue microarrays containing 90 cases PDAC and 60 cases corresponding adjacent normal tissue (Shanghai Outdo Biotech, Shanghai, China) were used to study NEK7 expression and the correlation with clinicopathological parameters. Use of all human samples was approved by the committee for ethical review of research involving human subjects at Peking University and Peking University Shenzhen Hospital, and informed consent was obtained from the patients.

### Immunohistochemistry and Evaluation

Tissues were sliced to sections of 4 μm. Endogenous peroxidase activity was blocked with methanol containing 0.3% hydrogen peroxidase. Antigen retrieval was performed by boiling in a microwave oven (citrate buffer, pH 6.0), as described before (29). Sections were incubated with antibody targeting NEK7, goat anti-NEK7(ab166776, Abcam) overnight at 4°C and stained with second antibody by using an SP-POD Kit according to the manufacturer’s instructions (#SP0041, Solarbio, China). Since there were no notable differences in NEK7 staining intensity, we evaluated the ratio of NEK7-positive pancreatic cancer cells. We counted the number of NEK7-positive cells among pancreatic cancer cells in at least 5 fields per section at 200× magnification. Samples were divided into NEK7-positive and NEK7-negative groups; NEK7-positivity was determined when the percent of NEK7-positive pancreatic cancer cells was greater than 5%. We investigated the correlation with NEK7-positivity with survival time, disease-free survival and clinicopathologic factors.

### Cell Lines, Culture Conditions, and Treatment

The following pancreatic cancer cell lines were used in this study: AsPC-1, CFPAC-1, Capan-2, KP-2, SW1990, Panc-1, BxPC-3, KP-3 and HPNE (Dr. K.Ohuchida, Department of Surgery and Oncology, Kyushu University, Fukuoka, Japan). All cell lines were maintained in Dulbecco’s modified Eagle’s medium (DMEM; Gibco, Grand Island, NY, USA), supplemented with 10% fetal bovine serum (Gibco), 100 U/mL penicillin, and 100 U/mL streptomycin (Invitrogen, Carlsbad, CA, USA) at 37°C in a humidified atmosphere with 5% CO_2_.

### Quantitative RT−PCR

Total RNA was extracted from cultured cells using a High Pure RNA Isolation Kit (Roche Diagnostics, Mannheim, Germany) and DNase I (Roche Diagnostics, Sigma-Aldrich) treatment according to the manufacturer’s instructions. RNA was directly reverse transcribed using the HiScript^®^ II 1st Strand cDNA Synthesis Kit (MR101-01, Vazyme, China) according to the manufacturer’s instructions. qRT-PCR was performed using the AceTaq^®^ qPCR SYBR Green Master Mix (Q121-03, Vazyme, China). For PCR analysis, three independent experimental repeats were performed, and data are presented as mean ± standard error of mean. We designed several specific primer sequences (Gene Pharma, China). Primer sequences are listed in **Table S1**. mRNA expression levels are presented as relative expression normalized to β-actin.

### Western Blot Analysis

Cells were prepared at 4°C in RIPA buffer (P0013J, Beyotime, China) containing proteinase inhibitor cocktail(B14001, bimake, USA) and phosphatase inhibitor cocktail (B15001, bimake). Proteins were separated on 4–20% precast mini polyacrylamide gels (SurePAGE™, Genescript, China) and transferred to PVDF membrane. Membranes were incubated overnight at 4°C with anti-NEK7 (ab95873, Abcam), anti-β-actin (66009-1-Ig, Proteintech, USA), anti-β-Tubulin (A12289, Abclonal, China). Membranes were then probed with appropriate secondary antibodies (Cell Signaling Technology). Immunoblot signals were detected by a Millipore chemical developer (Millipore Sigma, Burlington, MA, USA).

### Silencing of NEK7 Using Small Interfering RNAs

Gene silencing was achieved using small interfering RNA (siRNA, Gene Pharma). The following siRNAs directed against human NEK7 were used in the study: siRNA-1 (sense′, 5- GCAACUCAACCAUCCAAAUdTdT-3′; and siRNA-2 (sense, 5′-GAGGAUUGAGAUAAUCUAATT- 3′); GenePharma non-targeting siRNA served as a negative control. Cells were transfected with siRNA by using Lipofectamine™ 3000 Transfection Reagent (L3000015, Thermo Fisher) system according to the manufacturer’s instructions.

### Matrigel Invasion and Migration Assays

The invasiveness and migration capacity of cancer cells was assessed by determining the number of cells invading or migrating across transwell chambers as previously described (30). For invasion assays, cells (1 × 10^5^ cells/250 μl) were seeded in the upper transwell chamber (8 μm pore size; Becton Dickinson, Franklin Lakes, NJ, USA) containing 100 mL of reconstituted matrigel-coated membrane (20 μg/well, BD Biosciences, Bedford, MA, USA) at 24 h after knockdown of NEK7. Cells were incubated for 48 h and the number of invaded cancer cells was counted. Cell migration assays were performed using the same protocol as the invasion assay without a matrigel-coated membrane. Cells were allowed to migrate and counted 24 h after cell seeding into the upper chamber. In both assays and at each time point, invaded or migrated cells at the bottom of the chamber were fixed with 70% ethanol and stained with hematoxylin and eosin, and five random fields were counted at 100 × magnification (BX53, Olympus). Each experiment was performed in triplicate and repeated at least three times.

### Cell Viability Assay

Cells (1 × 10^3^ cells/well) were seeded in 96-well plates at 48 h after transfection with siRNA. Cell viability examined using the CellTiter-Lumi™ Plus Cell Viability Assay Kit (C0068M, Beyotime, China) following the manufacturer’s instructions. Background was subtracted using values from wells containing only culture medium.

### Adhesion Assay

The adhesion ability of PDAC cells was determined as previously described (31). Collagen I was used as the principal extracellular matrix molecule. Briefly, siControlor siNEK7 PDAC cells were labeled with CellTracker Green CMFDA (40721ES50, Yeasen, Shanghai, China) according to the manufacturer’s instructions. Then cells (4.0×10^4^/well) were added to the 96-well Collagen I coated plates, and cells were incubated for 3 h at 37°C. The plates were then washed three times with 200 µl of phosphate-buffered saline (PBS) to remove the non-adherent tumor cells. The number of adhered PDAC cells was determined in five random fields at ×100 magnification using a fluorescent microscope (Leica, DMi8, Germany). Each experiment was performed in triplicate and repeated at least three times.

### Establishment of Small Hairpin RNA and Luciferase−Expressing Cells

Two NEK7 small-hairpin (shRNA, shNEK7-1:ccggatatgggctataataca, shNEK7-2: ctccgacagttagttaatatg) vectors(Gene pharma) and a firefly luciferase expression vector (#LVP326; GenTarget, San Diego, CA, USA) were transfected into CFPAC-1 cells according to the manufacturer’s instructions. Non-targeting shRNA (Gene pharma) was used as control. Puromycin (S7417, Selleck Chemicals, USA) was used to select NEK7 or control shRNA-stably expressing clones, and blasticidin S hydrochloride (#15,205; Sigma-Aldrich) was used to select luciferase-expressing clones; selection was performed for more than 3 weeks. shRNA-mediated NEK7 knockdown was confirmed by quantitative RT-PCR and Westernblot.

### PDAC Organoid Culture

Organoids were established as described before (30). CFPAC-1 cells were embedded in growth factor-reduced Matrigel (Cat#356231; BD Bioscience, CA, USA), and cultured in human complete medium at 37 C° for 14 days (21, 25). Human complete medium was AdDMEM/F12(Cat#12634–010; Invitrogen, CA, USA), medium supplemented with 1M HEPES (Invitrogen), GlutaMax (Cat#35050–061; Invitrogen), penicillin/streptomycin (Cat#15140122; Invitrogen), B27 (Cat#17504044; Invitrogen), N-acetyl-l-cysteine (Cat#A9165; Sigma-Aldrich Co.), Wnt-3a (Cat#5036-WN-010; R&D Systems, MN, USA), R-Spondin1 (Cat#120–38; Peprotech, NJ, USA), Noggin (Cat#120-10C; Invitrogen), epidermal growth factor (EGF, Cat#AF-100-15; Peprotech), fibroblast growth factor (FGF, Cat#100–26; Peprotech), nicotinamide (Cat#N0636; Sigma-Aldrich Co.), Y-27263 (Cat# Y0503; Sigma-Aldrich Co.) and A83–01 (Cat#2939/10; R&D Systems).

### *In Vivo* Experiments

BALB/c athymic female nude mice were purchased (GemPharmatech, China) and transported to our institution at 4 weeks old. 10 Mice were randomized divided into two groups. After 1 week of accustomization, 1 ×10^6^ luciferase- and shRNA-stably expressing CFPAC-1 organoids were injected into the spleen of nude mice and liver metastasis was monitored and quantified every week by using the IVIS Spectrum. (Caliper Life Sciences, Waltham, MA, USA), after injecting 150 mg d-luciferin (#LK10000; Oz Biosciences, Marseille, France) into the intraperitoneal cavity of anesthetized mice. Luciferin emission was quantified using Living Image software, version 4.4 (Summit Pharmaceuticals International Corporation, Tokyo, Japan) until mice were sacrificed.

### Microarray

Total RNA was isolated from cultured cells using a High Pure RNA Isolation Kit with DNase digestion (Roche Diagnostics, Mannheim, Germany). RNA quality was evaluated using the Agilent 2200 TapeStation system (Agilent Technologies, CA, USA) for microarray analysis. RNA was labeled and hybridized to the Agilent SurePrint G3 Human Gene Expression Microarray 8 × 60K Ver.3.0 (Agilent Technologies). Data were analyzed using Feature Extraction software (Agilent Technologies).

### Database-Based Bioinformatics Data Mining

ICGC (International Cancer Genome Consortium, https://daco.icgc.org) gene expression data (32) of pancreatic cancer plus clinical data were obtained from ICGC website. ICGC-CA was the pancreatic cancer dataset from the Canada Pancreatic Cancer Genome Initiative. ArrayExpress gene expression dataset and corresponding clinical information of pancreatic cancer were downloaded from the ArrayExpress database at EMBL-EBI [24] under accession number E-MTAB-6134. The expression datasets of normal pancreas and pancreatic tumors used for the analyses described in this study were generated by the GTEx Portal (The Genotype-Tissue Expression Project, https://gtexportal.org/) and TCGA database (The Cancer Genome Atlas Program, https://www.cancer.gov/tcga). The datasets were arranged by UCSC Xena (33). The datasets were downloaded from UCSC Xena on 25 Sep 2019. The GTEx Project was supported by the Common Fund of the Office of the Director of the National Institutes of Health and by NCI, NHGRI, NHLBI, NIDA, NIMH, and NINDS. Bioinformatic analysis and statistical analysis were conducted using Perl language, R language and GraphPad prism.

### Statistical Analysis

The chi-squared test was performed to assess relationships between NEK7 protein expression and clinicopathological features. For results of *in vitro* experiments, values are expressed as the mean ± SEM. Comparisons of RT-QPCR were carried out using the Student’s t-test. Comparisons of RNA expression in bioinformatic analysis were performed using the Wilcox test and Kruskal-Wallis test. Kaplan–Meier analyses were compared using the log-rank test. P < 0.05 was used to define statistical significance. Statistical analysis was conducted using R language and GraphPad prism.

## Results

### NEK7 Overexpression Is Correlated With CD110-TPO Depletion in PDAC

**Figure 1:**
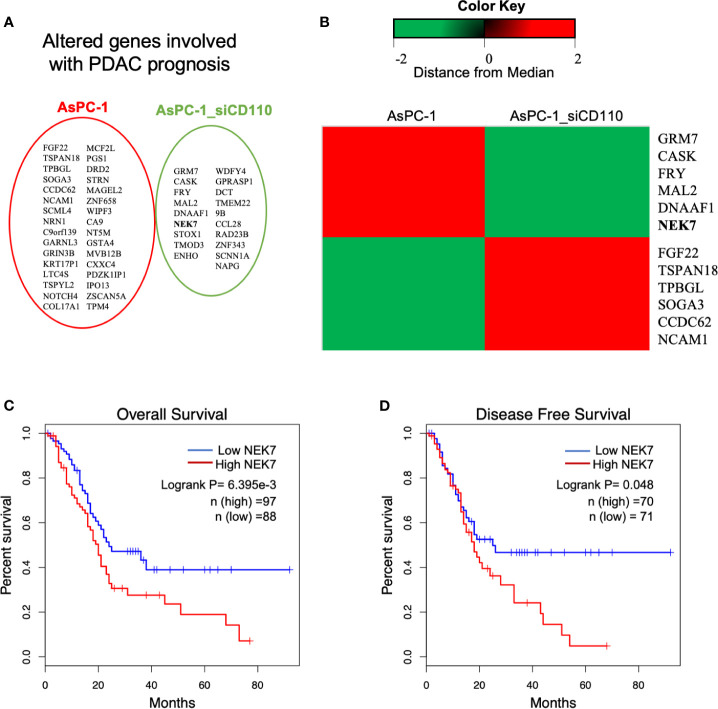
Analysis of microarray data revealed NEK7 correlated to TPO-CD110 axis. **(A)** Prognostic genes of PDAC were correlated with gene alteration of CD110 knockdown in TPO-CD110 axis. **(B)** Heatmap of top 12 genes significantly correlated to TPO-CD110 axis and PDAC prognosis. **(C)** Kaplan–Meier survival analysis of overall survival of PDAC patients according to NEK7 expression. NEK7-high expression was associated with shorter patient survival times (log-rank P = 6.395e-3). **(D)** Disease-free survival of PDAC patients according to NEK7 expression. NEK7-high expression was associated with shorter disease-free survival times (log-rank P = 0.048).

In previous study, we demonstrated the CD110-Thrombopoitin (TPO) axis promoted pancreatic cancer progression and liver metastasis by activating ERK1/2 and MYC (34). Then, a microarray analysis was carried out using AsPC-1 cells with CD110-intact or CD110-knockdown under stimulation of TPO. A heatmap of RNA expression profiles showed 632 differentially expressed genes in CD110- knockdown AsPC-1 cells under TPO stimulation. Survival analysis of these significant genes was performed using The Cancer Genome Atlas (TCGA) database (35, 36). We then screened 60 genes which was significantly correlated with survival outcome for patients with PDAC (**Figure 1A**). The top 6 up-regulated or down-regulated genes were arranged (**Figure 1B**). Among them, we selected NEK7 (Ratio: 0.1113796; Zscore: -2.11913071667481). Due to the expression of NEK7 significantly was correlated with both overall survival (**Figure 1C**; Logrank P=6.395e-3) and disease-free survival (**Figure 1D**; Logrank P=0.048) for pancreatic cancer patients.

### NEK7 Upregulation Is Correlated With Poor Prognosis in Patients With PDAC

**Figure 2:**
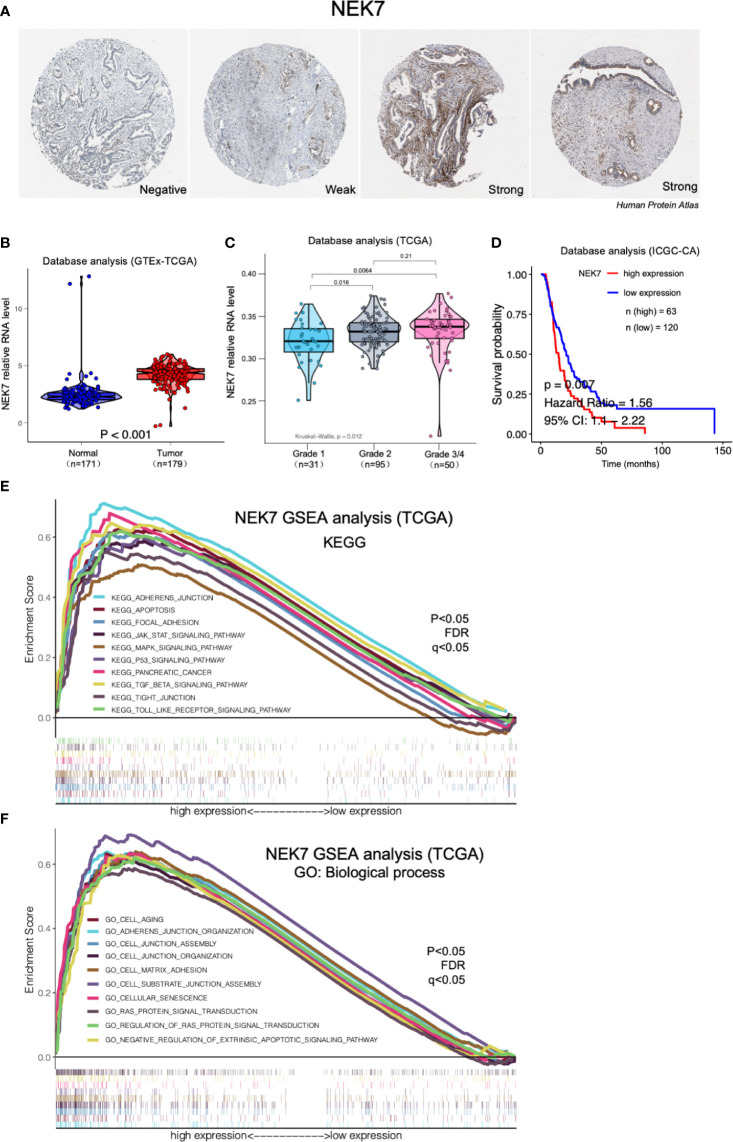
Expression of NEK7 correlated with pancreatic cancer progression in bioinformatic database. **(A)** Heterogenetic expression of NEK7 in pancreatic cancer tissues. **(B)** NEK7 expression was significantly upregulated in tumor tissues compared to tumor-adjacent normal tissues. **(C)** NEK7 expression in cancer cells was associated with poorly differentiated histological grade invasive ductal carcinoma. **(D)** Kaplan–Meier survival analysis showed NEK7-high expression was associated with shorter PDAC patient survival times in ICGC-CA database (log-rank P = 0.007). **(E, F)** GSEA analysis significant differences in enrichment of pathway changes in NEK7 high expression phenotype in KEGG and GO databases (p < 0.05, FDR q<0.05).

By analyzing Human Protein Atlas (available from www.protenatlas.org) (37), we observed that NEK7 expression was lower expressed in normal pancreatic ducts compared with tumor tissues; In primary tumor of PDAC, heterogenetic expression of NEK7 including negative, weak and strong was observed (**Figure 2A**). We found NEK7 expression was significantly upregulated in tumor tissues compared to tumor-adjacent normal tissues using bioinformatic analysis (**Figure 2B**); NEK7 overexpression was accompanied by poorly histological grade of PDAC (**Figure 2C**); Consist with our bioinformatic results, NEK7 upregulation was correlated with worse prognosis of patients with PDAC in ICGC (International Cancer Genome Consortium) database (**Figure 2D**). To further explore the role of NEK7, we conducted Gene Set Enrichment Analysis (GSEA) between low and high NEK7 expression samples, we found significant differences (p < 0.05) in enrichment of cell adherent, cell fate, and tumor microenvironment pathways in KEGG and Biological process (**Figures 2E, F**). The intersection of gene ontology terms showed that a proportion of differentially expressed genes was associated with cell-substrate junction, focal adhesion, and cell substrate adherens junction which might contribute to pancreatic cancer progression and invasiveness (**Figures S1A, B**). Moreover, we observed that NEK7 methylation status was negatively associated with histological grade of PDAC (**Figure S1C**); NEK7 hypermethylation was accompanied by favorable prognosis of PDAC survival (**Figure S1D**). Given the importance of NEK7 on PDAC progression, we suggest that NEK7 plays a crucial role in PDAC genomic alteration.

### NEK7 Is Overexpressed in Primary Tumors and Cell Lines of PDAC

**Figure 3:**
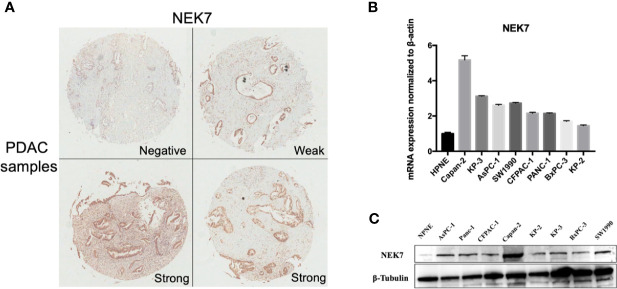
NEK7 expression in pancreatic cancer. **(A)** NEK7 expression was heterogeneity detected in pancreatic primary tumor. **(B)** NEK7 mRNA expression levels in pancreatic cancer cell lines and human pancreatic ductal epithelial cell (HPNE) cells. NEK7 mRNA expression was normalized by β-actin expression (p < 0.05). Error bar, error value in triplicate. **(C)** NEK7 protein expression level in pancreatic cancer cell lines and HPNE cells.

We examined NEK7 expression pattern using resected human tissues of normal pancreas and primary tumors from pancreatic cancer cases. NEK7 expression was heterogeneity observed in pancreatic primary tumor (**Figure 3A**). We further investigated NEK7 expression in human pancreatic ductal epithelial cell (HPNE) cells and pancreatic cancer cell lines. The qRT-PCR and western blotting results showed that NEK7 expression was slightly detectable in HPDE cells at both RNA and protein levels. The PDAC cell lines expressed NEK7 mRNA and protein at variable levels (**Figures 3B, C**).

### The Biological Function of NEK7 in PDAC *In Vitro*

**Figure 4:**
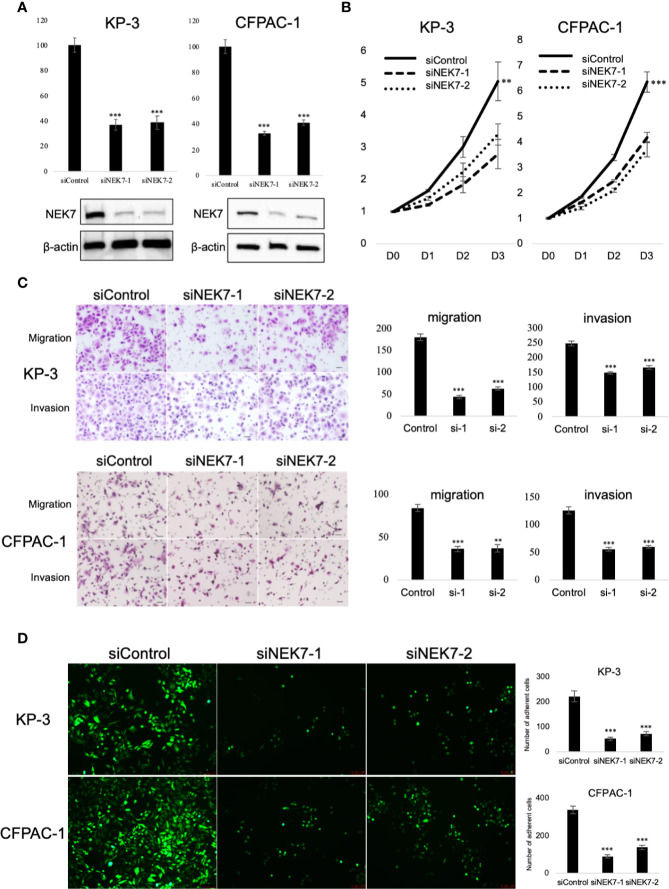
Knockdown of NEK7 reduces migration, invasion, cell viability, and cell adhesion of pancreatic cancer cells. **(A)** qRT-PCR (upper) and western blot (bottom) of NEK7 mRNA and protein levels in cells transfected with siRNAs targeting NEK7 or negative control. ***P < 0.001. **(B)** Cell viability of cancer cells was determined by CellTiter-Lumi™ Plus cell viability assay after 48h of NEK7 knockdown. **P < 0.01, ***P < 0.001. **(C)** Cells were transfected with the indicated siRNAs for 48 h, migration and invasion assays were performed for 24 and 48 h. Graphs show the quantification of cells calculated from five fields. Original magnification:100×. Scale bars = 100 μm. **P < 0.01, ***P < 0.001. **(D)** PDAC cells were dye with Cell Tracker green and examined for adhesion ability to collagen (I) Graphs show the quantification of cells calculated from five fields. Original magnification, ×100; ***P < 0.001.

To explore the biological role of NEK7 in PDAC cells, we investigated the effect of NEK7 knockdown on the invasiveness and migration activities of PDAC cell lines. We chose CFPAC-1 and KP-3 cells due to their overexpression of NEK7 and GSDMD (**Figure S3B**). Downregulation of NEK7 expression in CFPAC-1 and KP-3 cells were achieved stably and efficiently by using RNA interfering technique (**Figure 4A**). Cell proliferation was significantly decreased following downregulation of NEK7 expression (**Figure 4B**). Meanwhile, the migration and invasion activities of PDAC cells were significantly decreased after NEK7 knockdown compared to control group (**Figure 4C**). Next, we assessed the adhesion ability of PDAC cells to Collagen I and found that the adhesion ability of PDAC cells was significantly decreased following NEK7 downregulation (**Figure 4D**).

### Downregulation of NEK7 Suppressed PDAC Liver Metastasis *In Vivo*

**Figure 5:**
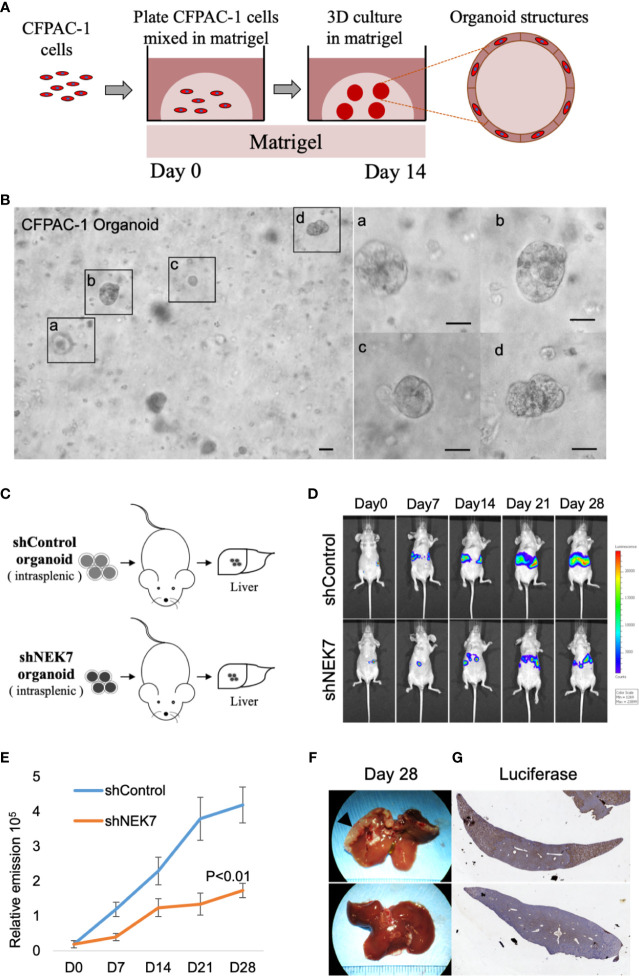
NEK7 decreased liver metastasis formation in xenograft organoid model. **(A)** Protocol for growing pancreatic cancer cells in 3D Matrigel assay (see Materials and Methods). **(B)** Microphotograph of CFPAC-1 derived cancer organoid. Scale bars =100 μm. **(C)** Scheme of xenograft experiment. Female nude mice were intrasplenic transplanted with cancer organoids and randomized divided into 2 groups (n = 5/group). 4 weeks after implantation, mice were sacrificed and liver metastases were harvested. **(D, E)** Liver metastasis was monitored and quantified weekly for 4 weeks using bioluminescence imaging. Mice injected with organoids expressing shNEK7 showed significantly reduced liver metastasis as observed by decreased luciferase activity. P < 0.01. **(F)** Gross pathology showed that knockdown of NEK7 significantly reduced liver metastasis formation (arrowheads: metastasis lesions). **(G)** Luciferase activity revealed decreased emission value in livers of shNEK7 xenograft mice.

We next investigated the effect of NEK7 downregulation on liver metastasis in the mice model. Our previous report showed that pancreatic tumor organoid recapitulate the histology and gene expression of its parental tumor (30). We therefore generated cancer organoids for 2 weeks using Luciferase-expressing CFPAC-1 cells transfected with control shRNA or NEK7 shRNA (**Figure 5A**). Established organoids (**Figure 5B**) were then splenic transplanted into nude mice and evaluated the tumor growth using IVIS system every week for 28 days (**Figure 5C**). Liver metastases from mice injected with NEK7-depleted cells showed significantly reduced luciferase activities compared to control group in which only fewer numbers of metastases occurred (**Figures 5D–F**). Compared to control group, mice with NEK7-depleted cells exhibited decreased liver weight and volume, but the differences were not significant (data not shown). Moreover, histological analysis using luciferase assay revealed that cancer cell metastatic into liver parenchyma was suppressed in mice injected with NEK7 knockdown cancer organoids (**Figure 5G**), indicating that downregulation of NEK7 in CFPAC-1 organoids suppressed liver metastasis capacity. Taken together, these results indicate that downregulation of NEK7 reduced colonization and proliferation of pancreatic cancer cells and inhibited liver metastasis formation *in vivo*.

### Correlations Between NEK7 Expression and Clinicopathological Features in PDAC

**Figure 6:**
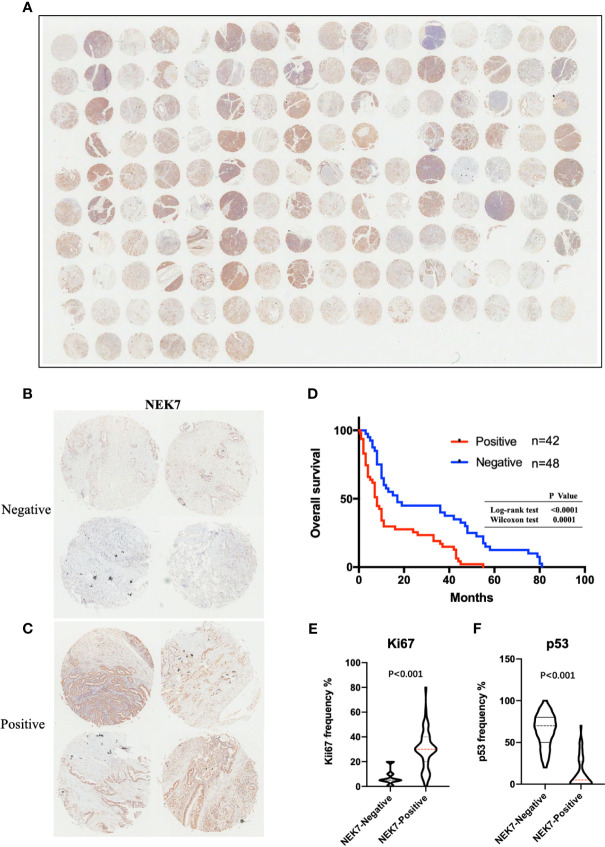
NEK7 expression correlates with poor survival in PDAC. **(A)** Image of NEK7 expression on human tissue microarray. **(B, C)** Representative four images of NEK7-negative pancreatic tumors (<5% cells expressing NEK7) and NEK7-positive pancreatic tumors (≥ 5% cells expressing NEK7). **(D)** Kaplan–Meier survival analysis of overall survival of pancreatic cancer patients according to NEK7 expression. NEK7-positive expression was associated with shorter patient survival times (log-rank test, P < 0.0001; Wilcoxon test, P = 0.0001). **(E, F)** Positive NEK7 expression was correlated to high Ki67 expression and low p53 expression in PDAC tissues. p < 0.001.

We then investigated NEK7 expression pattern in tissues resected from 90 patients with pancreatic cancer using microarray analysis and immunohistochemical analysis (**Figure 6A**). We then evaluated the correlation between NEK7 expression and clinicopathological features. We divided the pancreatic cancer patients into two groups: NEK7-negative group (NEK7 positive cancer cells < 5%; n = 48, **Figure 6B**) and NEK7-positive group (NEK7 positive cancer cells ≥ 5%; n = 42, **Figure 6C**). Patients in the NEK7-positive group had more frequent advanced T stage (P = 0.0485), lymphatic invasion (P = 0.0323) and higher histological grade (P = 0.0037) than patients in the NEK7-negative group in PDAC (**Table 1**).

**Table 1:** Correlations between NEK7 expression and clinicopathologic characteristics.

Characteristics	NEK7 expression		P value
	Positive (n=42)	Negative (n=48)	
Age			0.3561
≥65	27 (64.3%)	31 (64.6%)	
<65	15 (35.7%)	17 (35.4%)	
Gender			0.4180
Female	18 (42.9 %)	20 (41.7%)	
Male	24 (57.1 %)	28 (58.3%)	
Size			0.3340
≤5cm	25 (60.0 %)	32 (66.7 %)	
>5cm	17 (40.0 %)	18 (33.3 %)	
T stage			0.0485
T1/T2	10 (23.8 %)	9 (18.8 %)	
T3	32 (76.2 %)	39 (81.2 %)	
N stage			0.0527
N0	15 (35.7 %)	20 (41.7 %)	
N1	27 (64.3 %)	28 (58.3 %)	
M stage			NA
M0	42 (100 %)	48 (100 %)	
M1	0 (0 %)	0 (0 %)	
AJCC stage			0.1872
I	17 (40.5%)	22 (45.8%)	
II	25 (59.5%)	25 (52.1%)	
III/IV	0 (0%)	1 (2.1%)	
Histologic grade			0.0037
G1/G2	12 (28.6%)	19 (39.6%)	
G3	30 (71.4%)	29 (60.4%)	
Lymphatic invasion			0.0323
Negative	3 (7.1%)	5 (10.4%)	
Positive	39 (92.9%)	43 (89.6%)	
Vascular invasion			0.3738
Negative	10 (23.8%)	18 (37.5%)	
Positive	32 (76.2%)	30 (62.5%)	
Perineural invasion			0.2370
Negative	14 (33.3%)	16 (33.3%)	
Positive	28 (66.7%)	32 (66.7%)	

### NEK7 Contributes to Prognosis of PDAC

Furthermore, we investigated the correlation between NEK7 expression and overall survival and disease-free survival of the 90 pancreatic cancer patients with surgical resection. NEK7-positive patients accompanied by worse survival than NEK7-negative patients (P = 0.0030; **Figure 6D**). The median survival was 11.7 months for the NEK7-positive group and 18.2 months for the NEK7-negative group. Univariate analysis revealed that positive NEK7 expression (P < 0.0030), histologic grade (P < 0.0274), and positive lymphatic invasion (P = 0.0175) were all associated with overall survival (**Table 2**). We next performed multivariate analysis based on the Cox proportional hazard model using all parameters that were significantly associated with survival by univariate analysis. Multivariate analysis showed significant independent prognostic values in NEK7 positivity (relative risk 2.175; P = 0.0245) and positive lymphatic invasion (relative risk 2.924; P = 0.0252; **Table 3**). Moreover, we confirmed that expression of NEK7 was significantly increased in tumor tissues than tumor-adjacent normal tissues in patients with PDAC (P = 0.0252; **Table 4**). Positive NEK7 expression was significantly correlated with higher Ki67 expression and lower p53 expression (P< 0.001; **Figures 6E, F**).

**Table 2:** Univariate survival analysis of conventional prognostic factors and NEK7 expression.

Characteristics	Number of cases	Median OS (months)	P value
NEK7 expression			0.0030
Positive	42	11.7	
Negative	48	18.2	
Age			0.9324
≥65	58	19.5	
<65	32	32.3	
Gender			0.3627
Female	38	15.4	
Male	52	17.9	
Size			0.235
≤5cm	55	18.6	
>5cm	35	17.4	
T category			0.0725
T1/T2	19	32.7	
T3	71	12.3	
N category			0.0827
N0	35	32.4	
N1	55	15.2	
AJCC stage			0.3721
I	9	40.0	
II	55	26.1	
III/IV	26	9.0	
Histologic grade			0.0274
G1/G2	31	26.6	
G3	59	9.9	
Lymphatic invasion			0.0175
Negative	8	32.4	
Positive	82	14.7	
Vascular invasion			0.5673
Negative	28	32.3	
Positive	62	14.2	
Perineural invasion			0.4644
Negative	30	36.2	
Positive	60	16.4	

**Table 3:** Multivariate analysis of conventional prognostic factors and NEK7 expression.

Characteristics	Relative risk	95% confidence interval	P value
Positive NEK7 expression	2.175	0.880-3.194	0.0245
Low histologic grade	1.724	0.475-5.688	0.5243
Positive lymphatic invasion	2.924	0.663-6.815	0.0252

**Table 4:** Differential expression of NEK7 in cancer and adjacent tissues.

	n	NEK7 expression		Chi-square Value	p value
		Positive	Negative		
Pancreatic cancer	60	29 (48.3%)	31 ( 51.7%)	16.172	0.0045
Adjacent tissues	60	14 (23.3%)	46 (76.7%)		

## Discussion

In our study, we examined NEK7 expression pattern in PDAC and investigated its functional effect on pancreatic cancer progression. Our results showed that NEK7 was widely detected in PDAC cell lines and associated with the migratory, invasive, proliferation, and adherent capacities of cancer cells. *In vivo* experiments using a cancer organoid splenic injection model revealed that downregulation of NEK7 inhibited cancer cell liver metastases. Moreover, immunohistochemical analyses showed NEK7 expression was significantly correlated with advanced T stage, poorly histological grade and lymphatic invasion. Therefore, NEK7 might be a predictive marker of poor prognosis of PDAC. Taken together, these results suggest the potential of NEK7 being a evaluated biomarker for PDAC progression and liver metastasis.

Previous studies showed overexpression of NEK7 and NEK6 in a series of human tumor types, and the biological roles of NEK6 and NEK7 were extremely similar in cell cycle and cell proliferation enhancement (12, 27). We conducted gene ontology terms analysis of NEK6 in normal tissues and tumor tissues, the results showed that a proportion of differentially expressed genes was associated with focal adhesion, cell substrate adherens junction, cell-substrate junction which was consistent with NEK7, and Ada2/Gcn5/Ada3 transcription activator complex differ from mitochondrial protein complex and mitochondrial inner membrane of NEK7 (**Figures S2A, B**). Unlike NEK7, we did not observe a significant correlation between NEK6 expression and prognosis of PDAC patients by analyzing TCGA database (**Figure S2C**). Furthermore, we found no significant correlation between NEK7 and NEK6 expression in PDAC tumor tissues (**Figure S2D**). According to our results, although NEK7 and NEK6 are similar in structure, which contribute a similar function on enhancement of cell cycle, cell proliferation through mitosis and cytokinesis (38), their functions were not identical in PADC. In particular, recent studies proposed that ROS, potassium efflux and other factors activate Caspase-1 by regulating the interaction of NEK7 and NLRP3 inflammasome, leading to pyroptosis (39–41). We confirmed co-expression of NEK7 and GSDMD in cancer cells of PDAC tissues (**Figure S3A**). Meanwhile, we detected GSDMD protein on NEK7-positive PDAC cell lines (**Figure S3B**). These findings suggested a relationship between NEK7 and pyroptosis occurred in PDAC. However, there is no evidence of NEK6 relationship to pyroptosis in any cancer type. Given these findings, we speculate the difference in amino acid sequence between NEK7 and NEK6 may be a key factor in promoting the development of PDAC. Figuring out these differences will further uncover the underlying mechanism of PDAC progression.

Cell adhesion is one of the key mediators of cancer progression and metastatic dissemination. Cell adhesion molecules like integrins within the tumor microenvironment are changed and significant alterations are observed during cancer progression (38, 39). The changes of cell adhesion molecules alter the ability of tumor cells to interact with other cells and extracellular matrix proteins (40). Increasing evidences proved metastasis can be therapeutically targeted by blocking these cell-cell interactions (41, 42). Our results revealed NEK7 as a cell adhesion-related gene by bioinformatic analysis and biological experiment of Collagen I adhesion assay. Combining the results that NEK7 increased cell migration and invasion abilities *in vitro*, as well as increased liver metastasis formation *in vivo*. We hypothesized this might due to its upregulation of adhesion capacity. On the other side, our previous study proved TPO-CD110 axis promoted PDAC cell extravasation and liver metastasis by stimulating ERK1/2-MYC signaling pathway *in vitro* and *in vivo*. NEK7 was significantly involved in TPO-CD110 axis and PDAC prognosis. In PDAC, the particular mechanism of NEK7 involved in cell adhesion-mediated cancer progression and metastasis remains unclear.

In conclusion, positive NEK7 expression in PDAC was associated with poor prognosis of patients with PDAC. NEK7 upregulation increased PDAC cell proliferation and cell adherent capacities. NEK7 promoted PDAC cell invasiveness and metastasis *in vitro* and *in vivo*. These results suggest that NEK7 plays an important role in pancreatic cancer progression. Therefore, NEK7-targeted treatment might be beneficial for patients with PDAC.

## Data Availability Statement

The original contributions presented in the study are included in the article/**Supplementary Material**. Further inquiries can be directed to the corresponding author.

## Ethics Statement

The studies involving human participants were reviewed and approved by Ethics Committee of Human Experimentation at the Peking University Shenzhen Hospital. The patients/participants provided their written informed consent to participate in this study. The animal study was reviewed and approved by Animal Ethics Committee of Peking University Shenzhen Hospital.

## Author Contributions

ZY designed the study, conducted experiments, acquired and analyzed data, and wrote the manuscript. JQ analyzed bioinformatic data. ZFL, JY, YS, QL, and ZWL discussed and revised the manuscript. WY and GY conducted pathology analysis. JL and FL were responsible for the conception and supervision of the study and wrote the manuscript. All authors contributed to the article and approved the submitted version.

## Funding

This study was supported by Sanming Project of Medicine in Shenzhen (No. SZSM201612021); China Postdoctoral Science Foundation (No. 2020M682841).

## Conflict of Interest

The authors declare that the research was conducted in the absence of any commercial or financial relationships that could be construed as a potential conflict of interest.

## Supplementary Material

Supplementary Figure 1 Functional annotation of gene expression signatures between normal and tumor pancreatic tissues. **(A, B)** NEK7-related gene ontology terms. Genes that strongly correlated with NEK7 were screened by Spearman’s correlation analysis (Spearman |R| > 0.4, p < 0.05) based on the TCGA datasets. We then explored the biofunction of the NEK7-related genes by GO analysis. **(C)** Association between NEK7 DNA methylation status and pancreatic tumor histologic grade. The TCGA dataset was used for the analysis. **(D)** Kaplan–Meier survival curves for pancreatic cancer patients according to NEK7 DNA methylation status. The TCGA dataset was used for the analysis.

Supplementary Figure 2 Functional annotation of gene expression signatures between normal and tumor pancreatic tissues of NEK6. **(A, B)** NEK6-related gene ontology terms. Genes that strongly correlated with NEK6 were screened by Spearman’s correlation analysis (Spearman |R| > 0.4, p < 0.05) based on the TCGA datasets. We then explored the biofunction of the NEK6-related genes by GO analysis. **(C)** Kaplan–Meier survival curves for pancreatic cancer patients according to NEK6 expression. **(D)** Correlation of NEK7 and NEK6 in pancreatic cancer. The TCGA dataset was used for the analysis.

Supplementary Figure 3 NEK7 co-expressed with pyroptosis marker GSDMD in PDAC. **(A)** NEK7 co-expressed with GSDMD in cancer cells of PDAC tissues. **(B)** GSDMD protein was detectable on NEK7-positive PDAC cell lines.

## Abbreviations

PDAC, Pancreatic ductal adenocarcinoma; NEK, NIMA Related protein kinase; IHC, Immunohistochemical; qRT-PCR, Real‐time quantitative polymerase chain reaction; FBS, Fetal bovine serum; NC, Negative control; shRNA, Short hairpin RNA; TPO, Thrombopoietin; GTEx, The Genotype-Tissue Expression; TCGA, Cancer Genome Atlas Program; ICGC, International Cancer Genome Consortium; GSEA, Gene Set Enrichment Analysis; KEGG, Kyoto Encyclopedia of Genes and Genomes; GO, Gene ontology; BP, Biological process; GSDMD, Gasdermin D; OS, Overall survival; DFS, Disease free survival.
