# Supplementary figures and images for: NEK7 Promotes Pancreatic Cancer Progression And Its Expression Is Correlated With Poor Prognosis
Source: Front Oncol. 2021 Jul 6;11:705797. doi: 10.3389/fonc.2021.705797 (PMC8290842; doi:10.3389/fonc.2021.705797)

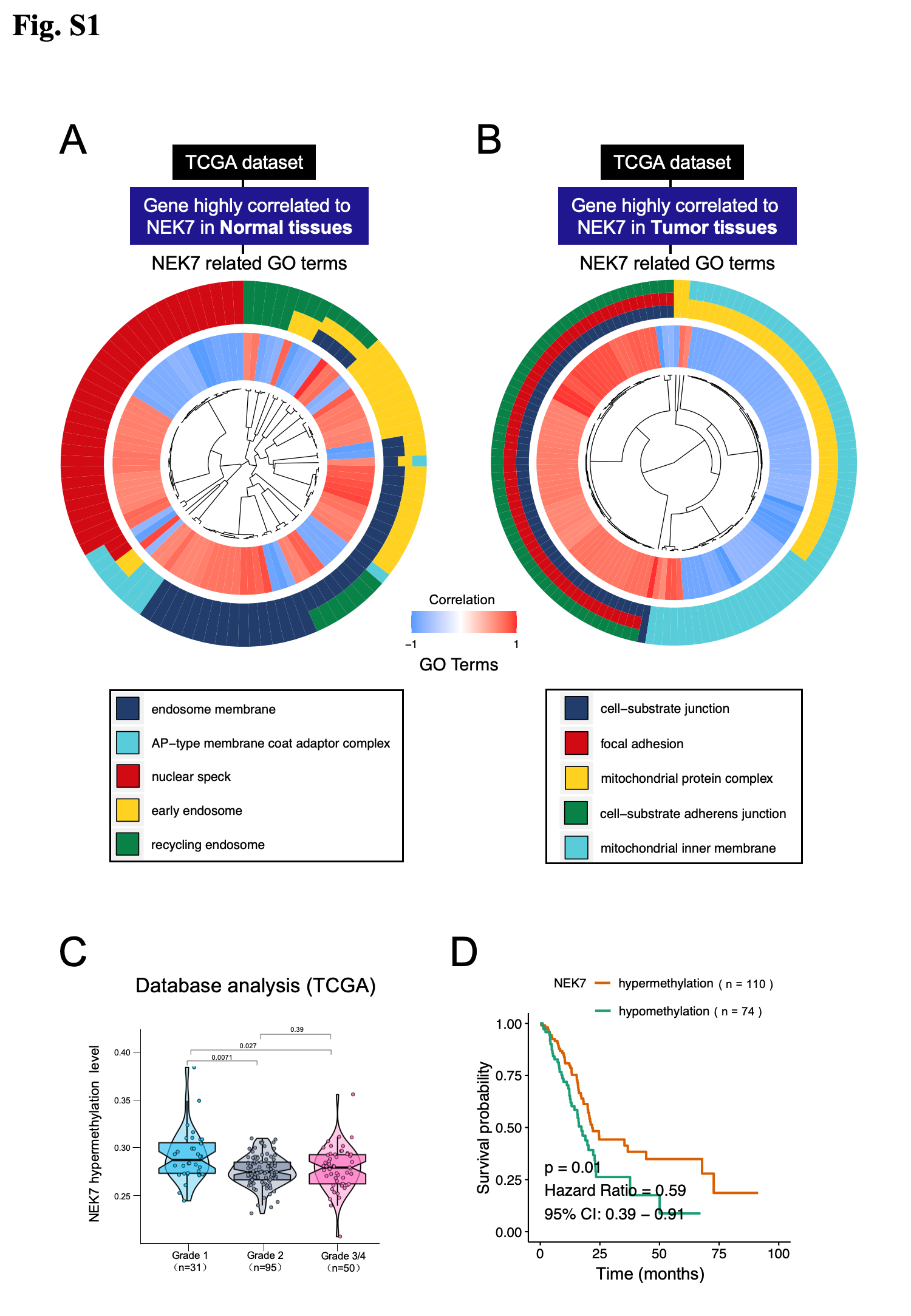

Supplement: Supplementary Figure 1 — Functional annotation of gene expression signatures between normal and tumor pancreatic tissues. (A, B) NEK7-related gene ontology terms. Genes that strongly correlated with NEK7 were screened by Spearman’s correlation analysis (Spearman |R| > 0.4, p < 0.05) based on the TCGA datasets. We then explored the biofunction of the NEK7-related genes by GO analysis. (C) Association between NEK7 DNA methylation status and pancreatic tumor histologic grade. The TCGA dataset was used for the analysis. (D) Kaplan–Meier survival curves for pancreatic cancer patients according to NEK7 DNA methylation status. The TCGA dataset was used for the analysis. [file Image_1.tiff]

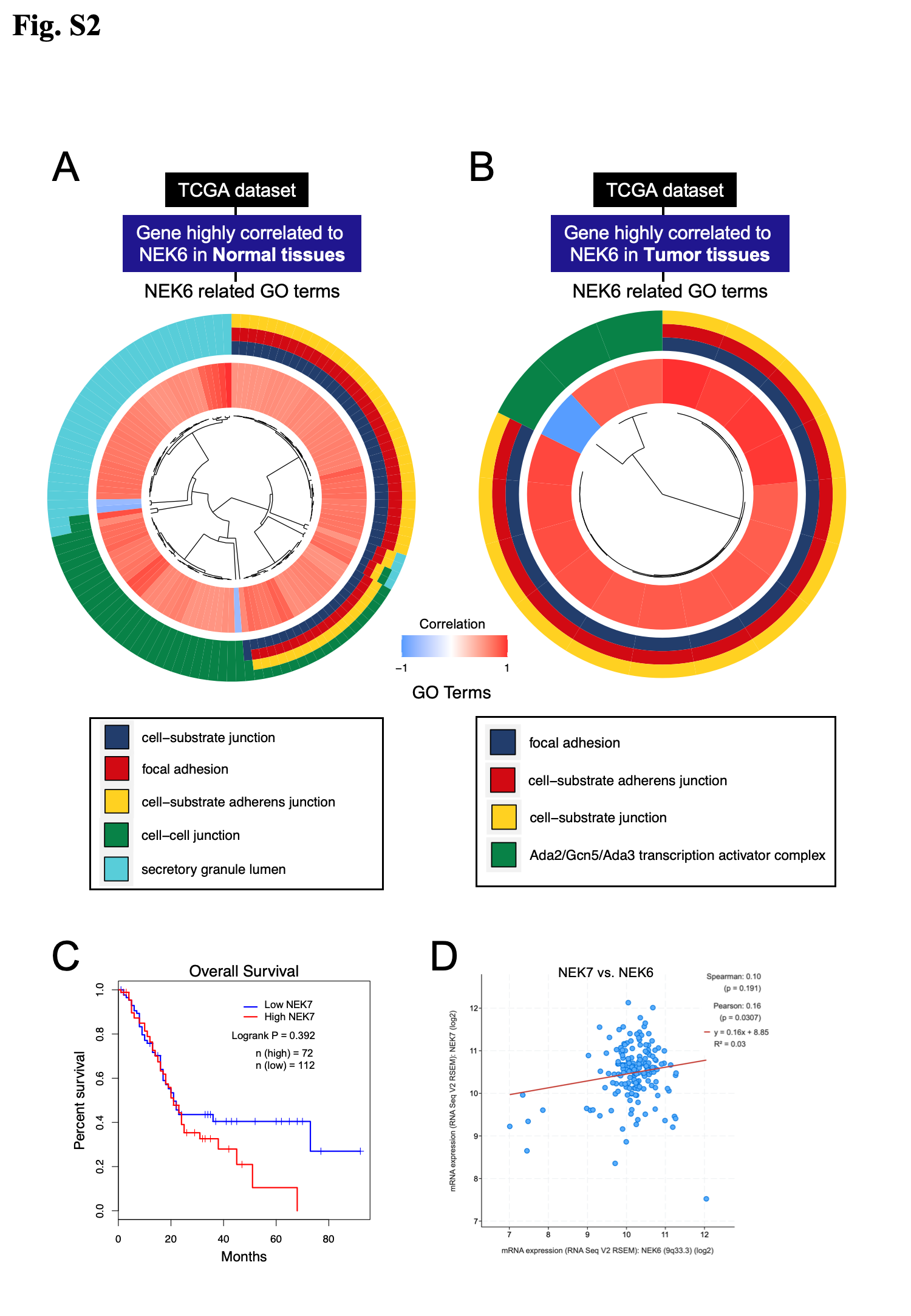

Supplement: Supplementary Figure 2 — Functional annotation of gene expression signatures between normal and tumor pancreatic tissues of NEK6. (A, B) NEK6-related gene ontology terms. Genes that strongly correlated with NEK6 were screened by Spearman’s correlation analysis (Spearman |R| > 0.4, p < 0.05) based on the TCGA datasets. We then explored the biofunction of the NEK6-related genes by GO analysis. (C) Kaplan–Meier survival curves for pancreatic cancer patients according to NEK6 expression. (D) Correlation of NEK7 and NEK6 in pancreatic cancer. The TCGA dataset was used for the analysis. [file Image_2.tiff]

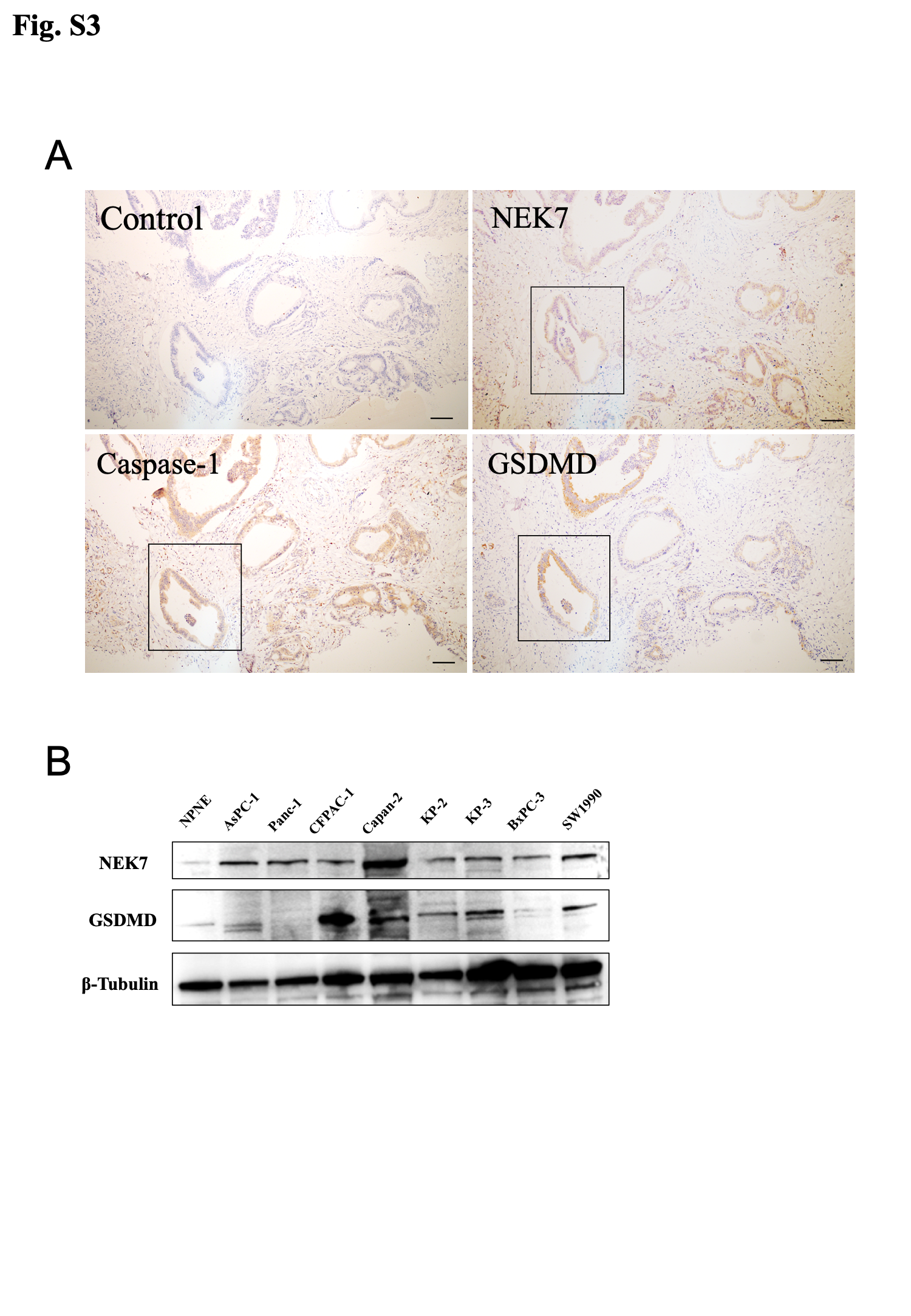

Supplement: Supplementary Figure 3 — NEK7 co-expressed with pyroptosis marker GSDMD in PDAC. (A) NEK7 co-expressed with GSDMD in cancer cells of PDAC tissues. (B) GSDMD protein was detectable on NEK7-positive PDAC cell lines. [file Image_3.tiff]
