# Supplementary material for: NEK7 Promotes Pancreatic Cancer Progression And Its Expression Is Correlated With Poor Prognosis
Source: Front Oncol. 2021 Jul 6;11:705797. doi: 10.3389/fonc.2021.705797 (PMC8290842; doi:10.3389/fonc.2021.705797)
Supplement: Supplementary file 4 [file Table_1.docx]

sense（5'-3'） antisense（5'-3'）

1.NEK7 primer (human): CACCTGTTCCTCAGTTCCAAC CTCCATCCAAGAGACAGGCTG

2.NEK7 primer (human): AGGCTTACTTGGTGACACACTGG CACCGTGCAGGTGACTCGAACC

3.NEK7 primer (human): CCGGAGAAGTGGAAATGGTGT CCAGACTATCAGTAACCCTCAAAGCC
